# Standing on the Shoulders of the Giants: Dr. Kanti R Rai

**DOI:** 10.46989/001c.121408

**Published:** 2024-07-22

**Authors:** Mohamad Mohty, Kanti Rai

**Affiliations:** 1 Sorbonne Université; 2 The Feinstein Institutes for Medical Research, Northwell Health, Manhasset, NY 11030

**Keywords:** Giants, chronic lymphocytic leukemia

**Figure 1.**
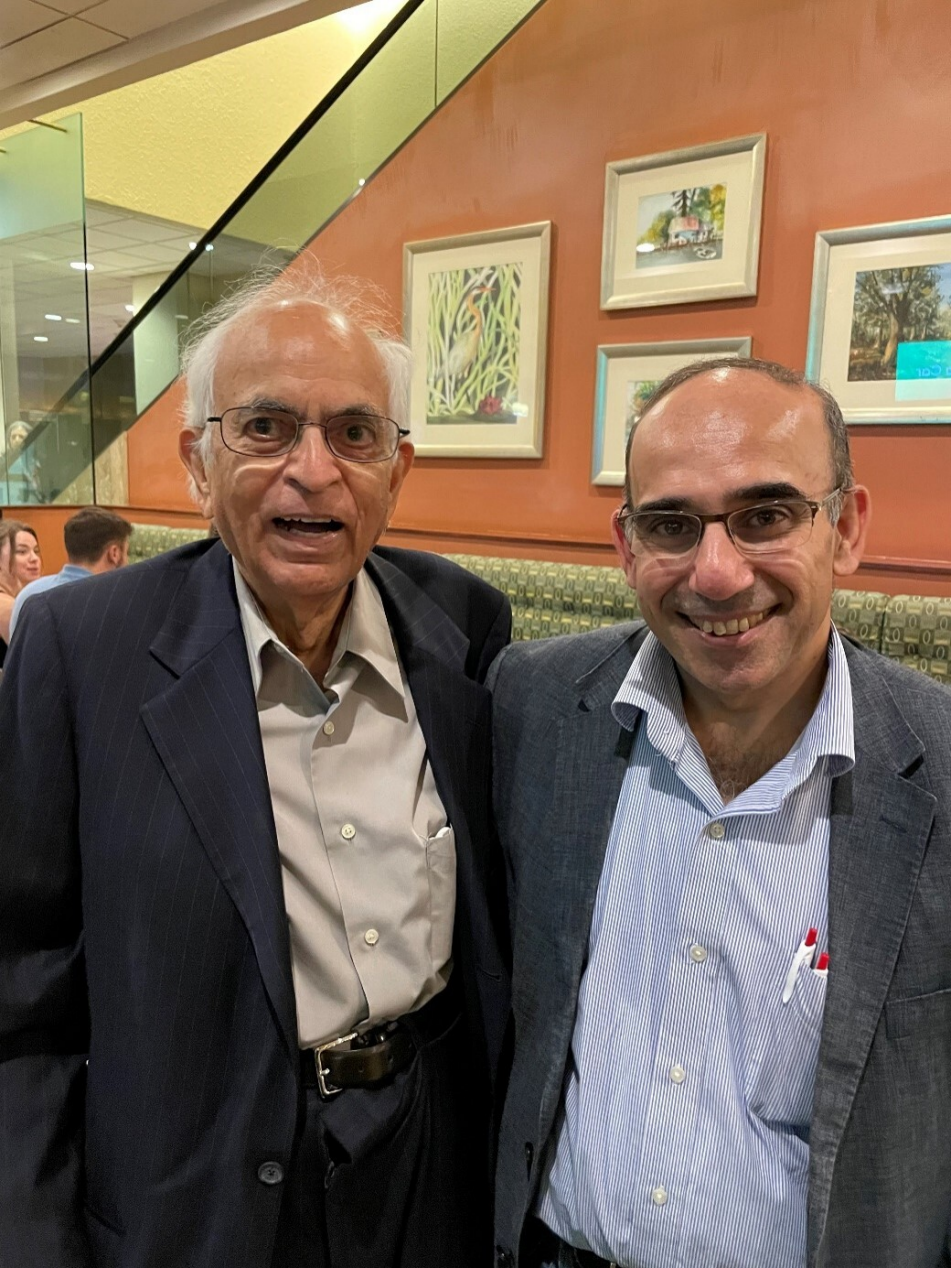
Dr Mohty and Dr Rai in December 2022.

In a letter to Robert Hooke in 1675, Sir Isaac Newton made his most famous statement: “if I have seen further, it is by standing on the shoulders of giants.” This initiative of the International Academy for Clinical Hematology (IACH) aims to celebrate the achievements of leading experts and investigators whose work and research have helped to significantly advance the field of clinical hematology and establish the milestones and foundations of modern clinical hematology. This report represents a transcript of the interview given by Dr Kanti R Rai (KR) (Figure 1) on the 8th of November 2022, who responded to a series of questions asked by Dr Mohamad Mohty (MM).

Dr Rai is the chief of Northwell Health’s CLL Research and Treatment Program. He is internationally recognized for his expertise in diagnosing and treating leukemia patients. He is a Joel Finkelstein Cancer Foundation Professor of Medicine, and Professor of Molecular Medicine at the Donald and Barbara Zucker School of Medicine at Hofstra/Northwell and is also Professor of Medicine at the Feinstein Institutes for Medical Research. He has been practicing now for more than fifty years. Dr Rai went to SMS Medical College in Jaipur, India and initially planned to become a pediatrician. He did his residency training in pediatrics, ending at North Shore University Hospital in 1959. He is board certified in pediatrics but ended up pursuing research in chronic lymphocytic leukemia (CLL) and became an expert in adult hematology. His ground-breaking research led to the establishment of the Rai Staging System for CLL and he has remained at the forefront of CLL research. His areas of interest include the natural history of CLL, how to improve prognostic markers of this disease and how to develop new strategies of therapy that are evidence- and science-driven. He has authored hundreds of peer reviewed articles and book chapters; he is an active member of the American Society of Hematology (ASH) and served as its president in 2006. Dr Rai is also a member of the American Society of Clinical Oncology (ASCO), and the American Association for Cancer Research. He is also a member of the International Workshop on CLL (iwCLL), CLL Global Research Foundation, and the Cancer and Leukemia Group B (now known as Alliance for Clinical Trials in Oncology) in the US. Dr Rai is the recipient of ASCO’s prestigious David Karnofsky Award (2012), iwCLL’s David Galton Award (2013), and ASH’s Wallace H. Coulter Award for Lifetime Achievement (2014).

MM: Good afternoon Dr Rai and thank you for joining us today. I wonder if you can tell us something about your early childhood and early educational experience, and how you ended up selecting your scientific interest in the medical field? You actually trained as a pediatrician and yet you ended up in CLL, a disease rather of the elderly patient.

KR: Yes, you are quite right that my original ambition and plan was to treat children, sick children with malnutrition and infections in rural Rajasthan, India. My uncle, my father’s brother, Dr Rai in Jodhpur, was a major influence on me and I remember as a child of six, seven or eight, when my uncle was in a village about 40 miles away from Jodhpur, in the summer vacation I would go and spend some time with my uncle. Every morning when I woke up, I would see outside our house a line of camels each with their driver, and I knew that they had been sent by people who had a sickness in their home and wanted the doctor to come and visit before he started his morning clinic. I was fascinated and once I asked my uncle if I could come with him. He said that I could but that he would go into the person’s house and I would promise to stay with the camel and camel driver and not cause any disturbance. I said I would do that and I rode up on the camel behind my uncle. This was exactly the same situation as in a Western town when a house visit was requested and a car and driver was sent to get the doctor. So, I was a good boy and did not cause any problems whilst my uncle was attending to the sick person. Word would go round that the doctor’s son was outside with the camel and the family would come out, take me inside and give me goodies- snacks, sweets, and take care of me. When my uncle was finished I would come back to the camel. This happened perhaps about four or five times that summer and I thought that being a doctor was not a bad thing at all, I get to ride the camel and to see a sick person and my children get rewards in return. I saw, during that summer, real malnutrition, and infections in that poverty-ridden little village near Jodhpur. I promised myself that when I grew up, I would train as a doctor and come back and take care of these sick children. Therefore, with a long answer to your short question, my early childhood really had a great influence in making my decision for me that when I grew up, I would become a doctor, a pediatrician in rural India.

MM: So, it was very successful, and you were indeed board certified in pediatrics. Can you tell us how you switched from pediatrics to CLL. Was there a particular event, incident, or faculty teacher that influenced this change in direction from sick children to the more chronic disease of older patients?

KR: The answer to your question is yes, yes, and yes! I did become a physician by going to the SMS Medical School in Jaipur, India from 1950 to 1955 and I got the degree in 1955 that in the US and France is called MD, and in India and Great Britain is called MBBS. As I had planned earlier, I still wanted to become a pediatrician and for further training in pediatrics I chose to come to the US. My first year was an assistant residency in pediatrics in a city hospital in the South Bronx of New York, called Lincoln Hospital. I stayed there for one glorious year from 1957 to 1958 and learnt a lot of clinical pediatrics. In 1958 I moved from the Lincoln Hospital to the North Shore Hospital in a suburb of New York called Manhasset. When I was chief resident in pediatrics here, I remember being on duty in August/September 1958, I was told that a new admission of a three-year old child was expected. I waited in the pediatric ward and then saw a young couple in the corridor leading from the pediatric ward, walking slowly and sadly. In front of them, this three-year old girl named Lori was jumping, not walking, from one foot to another, full of life. Behind her were her parents who seemed to have aged 20 years in the preceding two or three hours. When the child came to me, I introduced myself to the parents and started to take a history and found that this child was fine until the day before when she broke out in petechiae, skin blisters, and was fatigued. I examined her and then the attending physician came and introduced himself to me, a Dr Arthur Sawitsky. I told him I was the resident in pediatrics and Dr Sawitsky asked if I had ever made a diagnosis of acute leukemia in my career. I answered no, not a new diagnosis but at Lincoln Hospital I had seen two children with acute leukemia and I had taken care of them but unfortunately, both had died. He then said, “tonight you will make a new diagnosis”. He helped me do a bone marrow biopsy on this child and then made the specimen slides for the pathology department. He took me down to the laboratory and taught me how to stain the blood smears and the bone marrow aspirated smears and showed me those stained slides under the double-headed microscope. It was a fascinating and memorable teaching session. He also showed me, from the records, normal blood smears and bone marrow from three-year olds so that I could recognize these angry looking acute leukemia cells. Two or three hours of work in the laboratory showed me that we were dealing with acute leukemia of children. I asked Dr Sawitsky what we were going to do about Lori to which he replied that she would be dead within 6 to 18 months. I thought he was a heartless man who had no mercy or compassion. I was wrong, he was compassionate, but he was telling me of the true statistics of childhood acute leukemia in 1958. Not only did we lack a cure but hardly any child with acute leukemia had gone into complete remission, and indeed, Lori was readmitted two or three times in the next 8 months for complications and we treated her with the best-known methods of 1958. She died in her mother’s arms and I was affected so deeply by the experience, after bonding with this child who had been so full of life, that I realized that this was something I wanted to pursue. Dr Sawitsky would become my mentor and friend and said that I should come to the Long Island Jewish Medical Center, two miles from North Shore, and do a fellowship in hematology. I thought this was a good idea and I temporarily forgot about malnutrition and infectious diseases and in 1959 I went to do a fellowship in hematology and nuclear medicine. Dr Sawitsky continued to be my guide and mentor and I want to tell you that during your training, whoever becomes your friend, your mentor and teacher, can make a tremendous difference to what you become professionally. He suggested I do some leukemia research.

MM: Thank you very much for that amazing and very emotional story. Let me ask you about your research and in particular, what was the status of research in hematology and in CLL in the 60s, 70s and 80s when you started to become very active in the field after your training?

KR: My experience in 1958/1959 was in the primitive stages. In Boston and elsewhere in the US and in Europe, much activity was going on in the field of leukemia and as Siddhartha Mukherjee has said so effectively and eloquently in his book The Emperor of All Maladies, in which he starts the story of the history of cancer and the history of the development of effective treatments in acute childhood leukemia. I realized that that is exactly what the situation was, not only in childhood leukemia but in other hematological malignancies. We were, in the 60s and 70s, making empirical therapies, chemotherapies, and although work was going on, effective treatment of leukemia was truly absent.

At that time, I might note that at Burroughs Welcome (or Welcome), a pair of scientists called Gertrude “Trudy” Elion and George Hitchings, pharmacologists and chemists, were developing the first examples of what we now call alkylating agents: chlorambucil (or leukeran), busulphan (Myleran). Today they are what we call targeted therapies against cancer. Leukeran was developed in the 50s and by the 60s was commonly being used for CLL, and David Galton in London and William Dameshek in Boston had been using chlorambucil in CLL and it had shown good evidence of killing leukemic cells but had accompanying toxicity. So, we had some agents that we could use to treat malignancies but they were not good enough.

I am now over 90 years old and I can go back approximately 90 to 100 years and bring the history of hematology from 1924 when in Boston, Dr George Minot and Dr Raphael Isaacs published the first comprehensive paper on the clinical records of a large number of patients with CLL in the predecessor of the New England Journal of Medicine (The Boston Medical and Surgical Journal). Minot was later awarded the Nobel prize for Medicine, for similar work, and I recognize that Minot, Galton and Dameshek were ‘giants’ and it is on the shoulders of these heroes that made it possible for people like Kanti Rai to see further.

How I turned my attention from acute leukemia to CLL is another story which introduces my second mentor at the Brookhaven National Laboratory in Long Island, the giant, Eugene P. Cronkite, a hematologist. In 1958 my mentor suggested I do some leukemia research by going to Dr Cronkite at Brookhaven and in 1960, Dr Cronkite accepted me. This was the heyday of the study of bone marrow and peripheral blood leukemic or normal cell kinetics because thymidine, the precursor of DNA, had just been brought out as the radiolabeled isotope tritiated thymidine which can be used as a tracer and after giving this to the patient, bone marrow samples could be taken and autoradiography and beta particle emission were the technologies used.

I joined the Cronkite leukemia research team and learned first-hand the basic cell kinetics, the cell cycle time, DNA synthesis time etc. These were very labour intensive procedures 60 years ago, today, microscopy and autoradiography are done at the push of a button on a cell sorter and results of months of work performed with autoradiography can now be obtained instantaneously. I am not ashamed of the time taken to do this work which caused us excitement, exhilaration, and brought us knowledge. One day in Dr Cronkite’s clinic, I saw three different patients with CLL. One was attending once per year and was doing well; one was very sick and having frequent blood transfusions and infections and was being admitted practically every two weeks. The third CLL patient was somewhat in between these two extremes. After I had seen these three, Dr Cronkite came to discuss them and I said that we must be making some errors as these three CLL patients were very different from each other. Dr Cronkite put his hand on my shoulder and said, ” you are not making any error but that is the question I would like you to study and find an answer to, young man”. I did not think much more of this at the time and then a couple of weeks later this statement from Cronkite came to haunt me, I went into the chart library of Brookhaven and requested all the charts of the CLL patients that we had treated so that I could study them. There were about 40 and I summarized the histories, the lab results, and the clinical course of each one and because this was not my sole work, it took about a year. I put all of these patients’ charts on the three walls of my small cubicle and kept looking at them, sometimes changing the wall of a chart. I knew that Dr Galton and Dr Dameshek had been asking the same question as had Dr Wintrobe in Salt Lake City. I then placed all the charts of the patients who had died relatively quickly on one wall and all the patients who were living with CLL with no apparent complications, on the opposite wall, and all those in between on another. Bearing in mind we had no computers, only months and months of visually examining these chart summaries, but eventually, I saw the light and noticed that all the patients on one wall had low platelets and low haemoglobin at initial diagnosis which directed us towards clinical staging. I got a lot of credit for this observation but I want to give credit to Dr Cronkite and Dr Sawitsky and a lot of credit to Dr Jacques-Louis Binet at Pitié-Salpêtrière in Paris who, a few years after my observations, came up with a similar clinical staging system.

MM: What a fascinating story and with this in mind and once the staging system gained popularity, what were the interactions between all of these people you mentioned who were interested in the same topic, and what was the goal? It looks like there was a spontaneously common interest from people working in different places. As you said, the connections were not there, we couldn’t send an email and discuss things immediately. How did the field move forward?

KR: The field did move forward. All of these people were big people, Dameshek, Galton, Wintrobe and his team, but the more important thing was this simple method of classifying the disease empowered the practicing clinicians tremendously. They were now able to use a system by which they could be assured that they would not make a mistake, which made it possible for clinicians and researchers to start to ask pertinent questions in CLL which had not been possible previously as CLL comprised an amorphous group of different types of patients. This led to what my colleague Nicholas Chiorazzi in New York and in the UK, Freda Stevenson demonstrated, that the clinical staging is useful but that if the patient has the mutated IgHV (immunoglobulin heavy chain) gene on the leukemic lymphocytes, they will have a superior prognosis compared with those with unmutated status. Credit must go to Chiorazzi and Stevenson for finding this mutation, or lack of it, and Döhner in Germany doing fluorescence in situ hybridization (FISH) analysis, because karyotyping in CLL was unpredictable and impractical. Döhner and colleagues did FISH on chromosomes at risk and demonstrated that there is a hierarchy of cytogenetic abnormalities which predicts prognosis: 13q deletion had a better prognosis, 17p deletion had the worst prognosis. This type of work and the mutation of IgHV, the FISH cytogenetics, came 10 to 15 years after the clinical staging by Binet and by Rai.

What we are seeing today in CLL is that we do not have a cure but very predictable effective therapy to cause remission by targeting BTK (Bruton’s tyrosine kinase) inhibitors, BCL-2 inhibitors (B-cell lymphoma 2), which renders us a quality of remission which has not been seen except in a smaller population using chemoimmunotherapy developed by Michael Keating in Houston, and by Michael Hallek in Cologne, in a randomized trial. They made this progress in therapy become a reality in the subsequent decade.

MM: Thank you. Let me ask you about all the doctors, fellows, and students that you have trained as I think there are dozens and dozens of them across the globe, taking care of CLL patients and those with other hematologic malignancies. In the first part of this discussion you alluded to a mentor, especially the first one you meet. So did you see yourself playing a similar role in influencing these young physicians to go into the CLL field? What can you say about all these people you have trained?

KR: I have been very fortunate in having wonderful residents, fellows, and younger colleagues come to work with me and I am grateful to them. I do not have a super ego and feel that my mentors contributed to me immensely and I am sure I have influenced others but that is not for me to say. I do not consider myself a giant and so I would like to back away from this question because in my humility, it would be unlike me to tell you how many people I have mentored. Of course I have mentored, but was this the same kind of relationship that I had with Cronkite and Sawitsky? I don’t want to say that. Thank you.

MM: OK, well I still believe that you really are a true giant but let me move to another question. When I was preparing for this interview, I spent some time reading about you, looking at some of your publications, and I came across a sort of mantra that was on your ‘bio’. You wrote: “I’m grateful for the opportunity to serve my patients”. This brings me to the question on the relationship you had, and probably still have, with your patients. You told us about when you saw those three patients on the same day with different severities of disease and this was the trigger for you to answer the research question. How, in general, have patients influenced the research questions and the way that you have applied yourself in answering these questions?

KR: I have had a very unusually intimate relationship with a large proportion of my patients. Somehow, after several visits they have become my friends. They are fascinated by my way of thinking about their disease and their questions and we enjoy the one-to-one discussion about CLL and what I am learning from his or her disease. To the extent whereby, when some of them die, I become a part of their family. I visit them at home while they are sick; not everyone but quite a few. Your question on how does this drive my research questions? It is a mutual give and take and so it is not easy to tell you that at one particular point I may have changed my attitude about something. It is a conversation, an ongoing relationship which alters mine and the patient’s behaviour. Therefore, I think it is true that my research has certainly been guided by the patient.

MM: Impressive. You mentioned the word ‘family’ and ‘intimate relationship’ so can I push a little bit further and ask you to say a few words about the contribution of your family on your career. I heard about how your uncle’s story opened your eyes to medicine but what about the influence of your family on your career on a daily basis over the last few decades.

KR: My wife Susan, to whom I have been married for 54 years, has been a very big support and has been a very objective person. If she finds something not right I will trust her to be the first one to point it out and if I’m doing something right I will trust her to point it out to me. I have two children, Samantha, who is 53 and is a physician in family medicine. She and her husband Jay, also a physician, are also very supportive of me, giving me lots of credit while I fight and question whether they are right. My son Josh and his wife Cici are both very supportive. I have relatives, cousins, nephews and my siblings. My sister is 89 and obstetrician and gynecologist in Jaipur and has been a tremendous boost to my ego. All of them have made enormous contributions to what I became and what I am.

MM: Thank you. You are now over 90 Kanti, and are looking retrospectively at your career, so maybe this is a provocative question. Is there something that you think you would have done differently in hindsight?

KR: I do wonder that when I changed my course in 1958 and if I had not said ‘yes’ to Dr Sawitsky to become a fellow in hematology, what would have been the course? Going back to rural India and taking care of children was my original ambition and as I have always wanted to know ‘why’ and ‘how’ so I might have done the same in pediatrics and attained some success, or not as much success. That is a question that does come to me once in a while, but I am essentially a happy person with no regrets, no anger, no ‘what ifs’. Its not bad, at 90, to not self-satisfy but to be grateful for what I am.

MM: One last question if I may Kanti, what are your hopes for the future of hematology and maybe most importantly, you are a treasure for all of us. You are very humble but you are a big giant for us in the field of hematology. What is your advice for the younger generation?

KR: It’s a good question. I feel that the future of hematology is extremely bright. Whenever I notice that there is a combination of questions and actions at the clinical level combined with questions and work at the scientific technology level, when the two are combined we make logarithmic progress. Today, in 2023, we happen to be at that time when that combination is present, is occurring, on a technological basis, a bench-research questions basis, and a clinical observation and clinical question basis. When this combination occurs, we make tremendous progress. There was a period when there was one without the other and we went through decades without major achievements. Anyway, more than 100 years ago when Virchow discovered leukemia by autopsies and clinical observations, and Ehrlich came up with the technology of aniline dyes that we used to stain the blood and bone marrow samples, when the two combined, we made progress in understanding and diagnosing and we were talking about leukemia with the same questions. That was almost 200 years ago but that combination did not occur right away. Ehrlich developed the dyes and today we use the Wright-Giemsa stain and nobody questions who was Giemsa? who was Wright? I think it’s the same today, the clinical plus the technical; coincidence, at the same time, and I feel we will make progress.

MM: Thank you very much Kanti. I personally feel very honored, very privileged that you accepted to spend this hour or so with me in this interview. I would like to say a big thank you not only on behalf of all the hematological community but also on behalf of the many thousands of patients on whom you have had an impact.

